# Supplementary material for: Accurate Detection of Tandem Repeats from Error-Prone Sequences with EquiRep
Source: bioRxiv. 2024 Nov 7:2024.11.05.621953. Preprint. [Version 1] doi: 10.1101/2024.11.05.621953 (PMC11580891; doi:10.1101/2024.11.05.621953)
Supplement: Supplement 1 [file media-1.pdf]

Supplementary Materials for "Accurate Detection of Tandem Repeats from Error-Prone Sequences with EquiRep"

Zhezheng Song[1,†], Tasfia Zahin[1,†,], Xiang Li[1], and Mingfu Shao[1,2,*]

[1]Department of Computer Science and Engineering, School of Electronic Engineering and Computer Science, The Pennsylvania State University
[2]Huck Institutes of the Life Sciences, The Pennsylvania State University

October 25, 2024

List of Supplementary Figures

1 Comparison of number of correct predictions on simulated data at 20% error rate. 2
2 Comparison of number of instances with edits less than 10% of the unit length on simulated data at 20% error rate. 2
3 Comparison of average edit distance on simulated data at 20% error rate. 2
4 Comparison of number of correct predictions on simulations with 2 recurring kmers at 20% error rate. 3
5 Comparison of number of instances with edits less than 10% of the unit length on simulations with 2 recurring kmers at 20% error rate. 3
6 Comparison of average edit distance on simulations with 2 recurring kmers at 20% error rate. . . 3
7 Comparison of number of correct predictions on simulations with 3 recurring kmers at 10% error rate. 4
8 Comparison of number of instances with edits less than 10% of the unit length on simulations with 3 recurring kmers at 10% error rate. 4
9 Comparison of average edit distance on simulations with 3 recurring kmers at 10% error rate. . . 4
10 Comparison of number of correct predictions on simulations with 3 recurring kmers at 20% error rate. 5
11 Comparison of number of instances with edits less than 10% of the unit length on simulations with 3 recurring kmers at 20% error rate. 5
12 Comparison of average edit distance on simulations with 3 recurring kmers at 20% error rate. . . 5

List of Supplementary Tables

1 Comparison of running time in seconds fro different lengths and copy numbers on simulated data. 6

†These authors contributed equally to this work.
*Correspondence should be addressed to mxs2589@psu.edu.

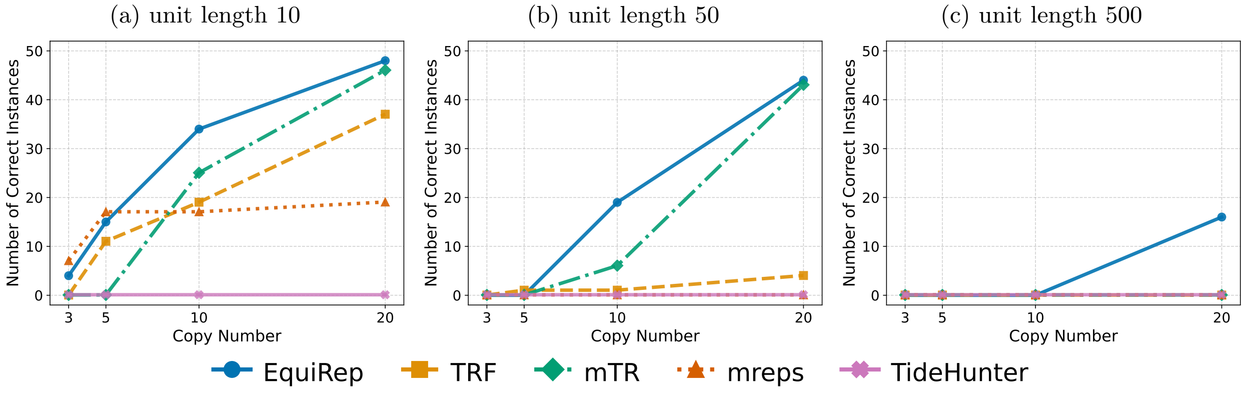

Supplementary Figure 1: Comparison of number of correct predictions on simulated data at 20% error rate.

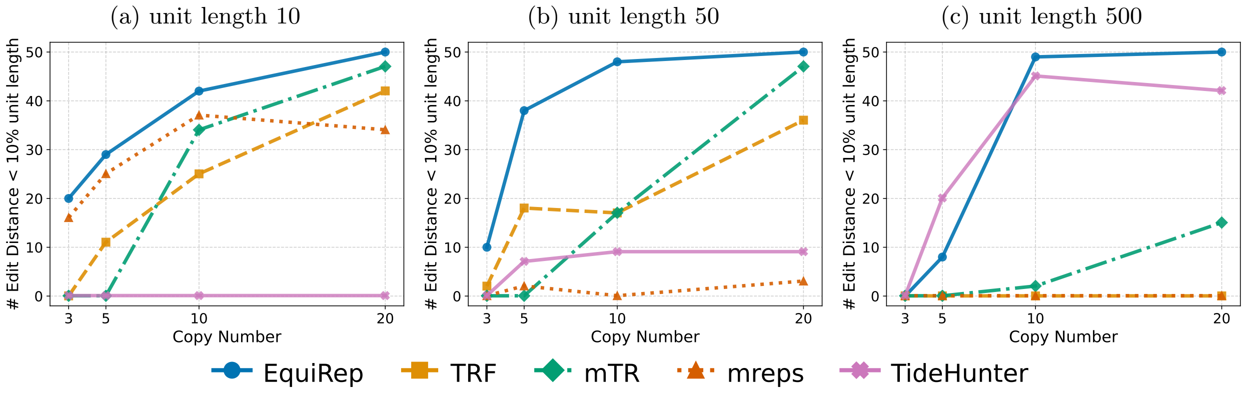

Supplementary Figure 2: Comparison of number of instances with edits less than 10% of the unit length on simulated data at 20% error rate.

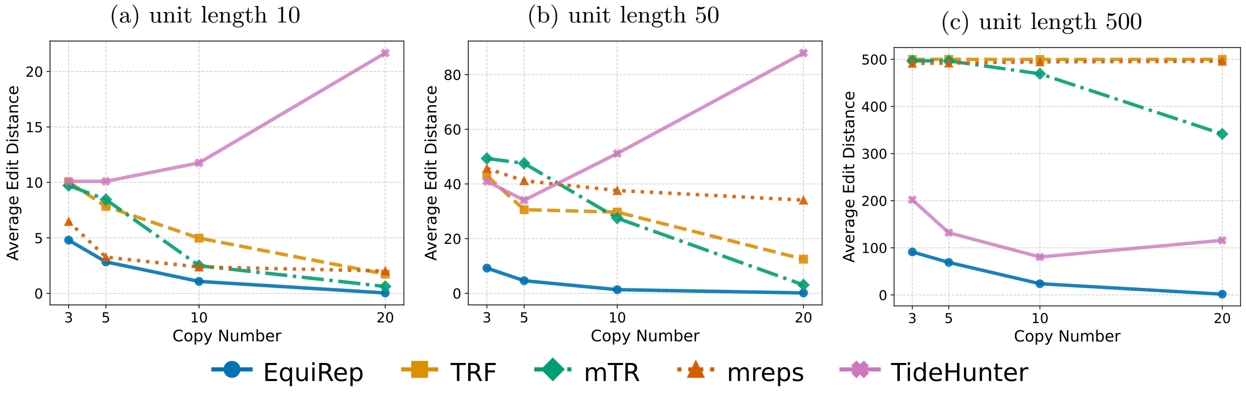

Supplementary Figure 3: Comparison of average edit distance on simulated data at 20% error rate.

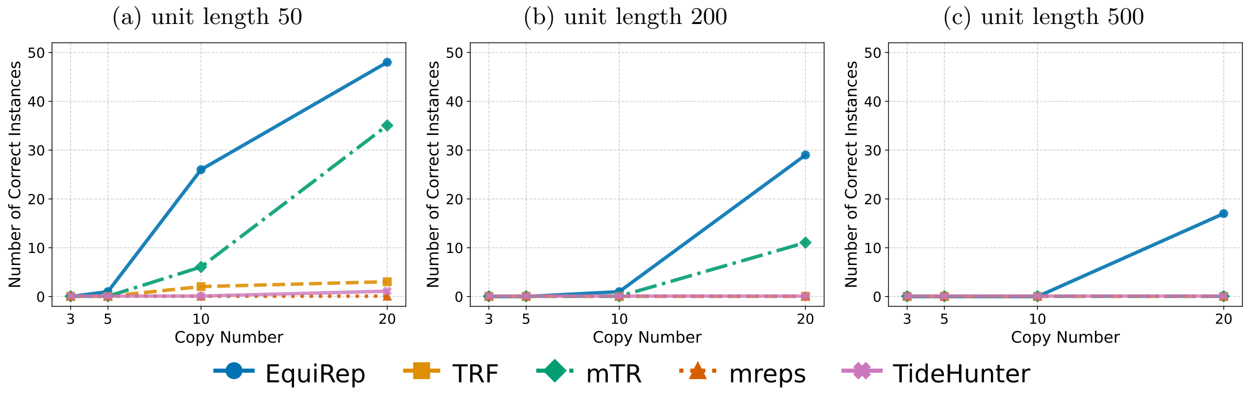

Supplementary Figure 4: Comparison of number of correct predictions on simulations with 2 recurring kmers at 20% error rate.

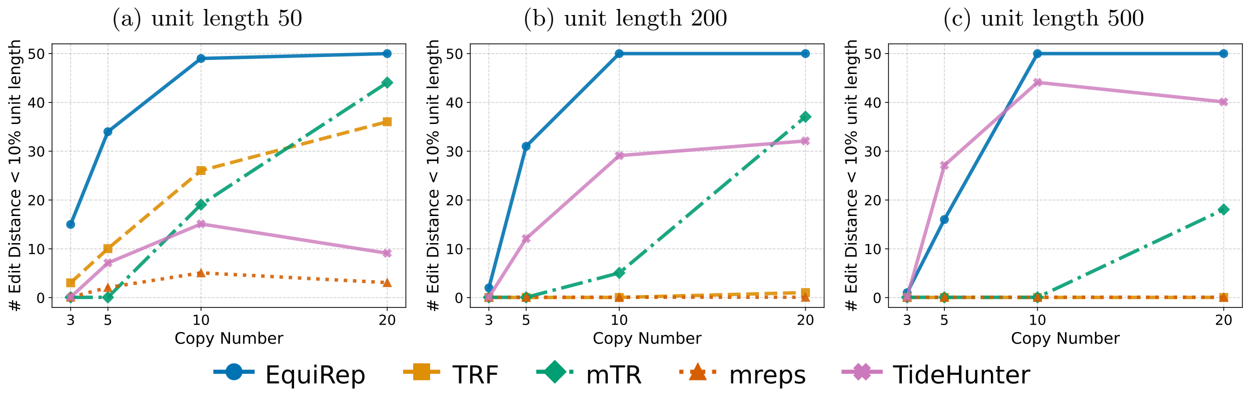

Supplementary Figure 5: Comparison of number of instances with edits less than 10% of the unit length on simulations with 2 recurring kmers at 20% error rate.

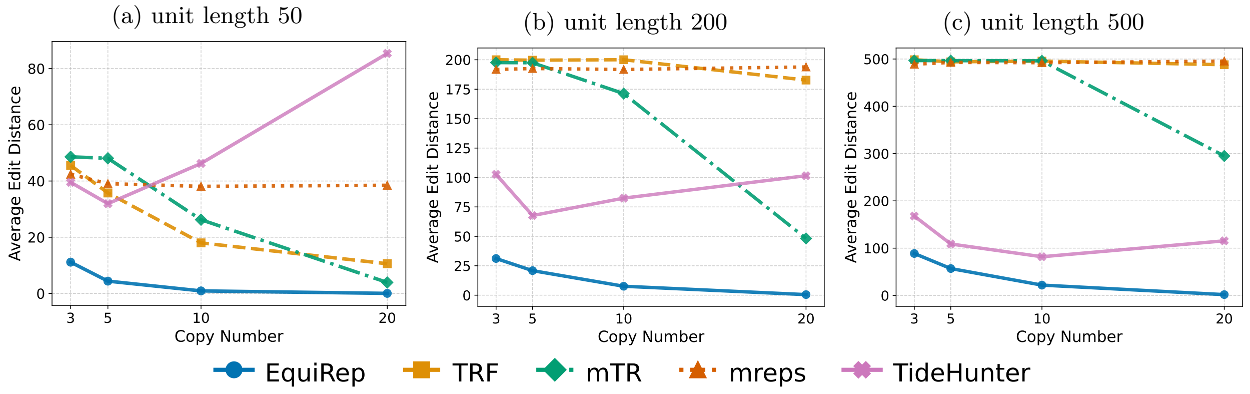

Supplementary Figure 6: Comparison of average edit distance on simulations with 2 recurring kmers at 20% error rate.

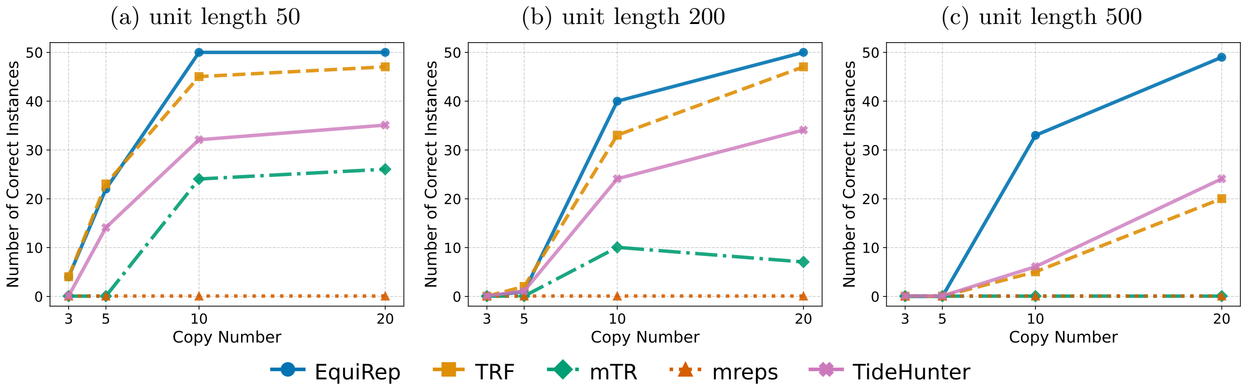

Supplementary Figure 7: Comparison of number of correct predictions on simulations with 3 recurring kmers at 10% error rate.

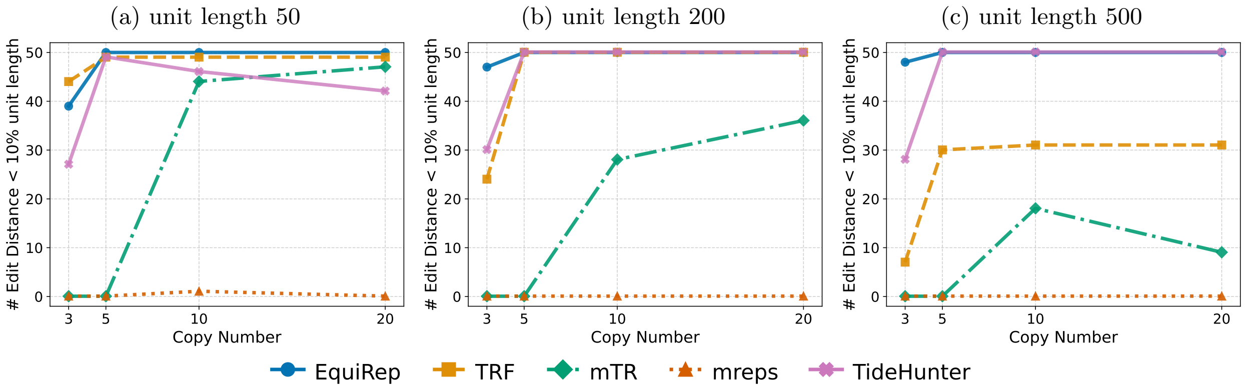

Supplementary Figure 8: Comparison of number of instances with edits less than 10% of the unit length on simulations with 3 recurring kmers at 10% error rate.

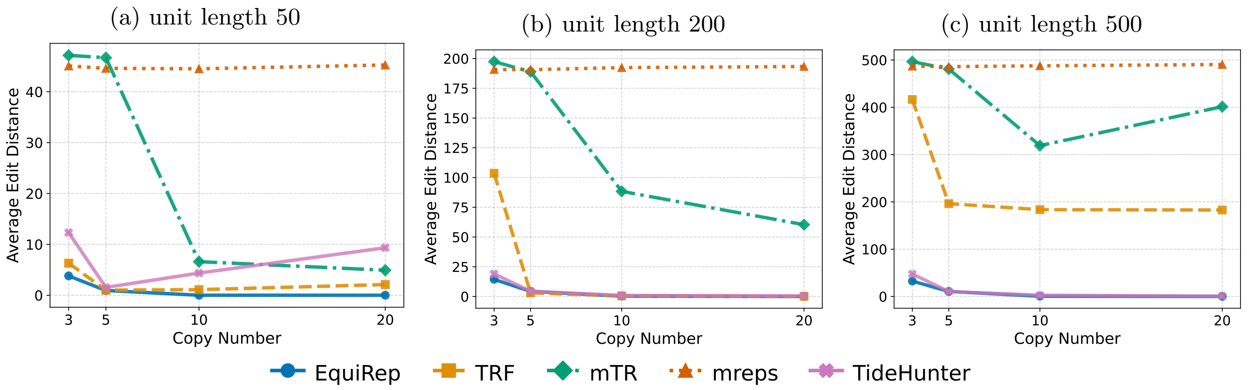

Supplementary Figure 9: Comparison of average edit distance on simulations with 3 recurring kmers at 10% error rate.

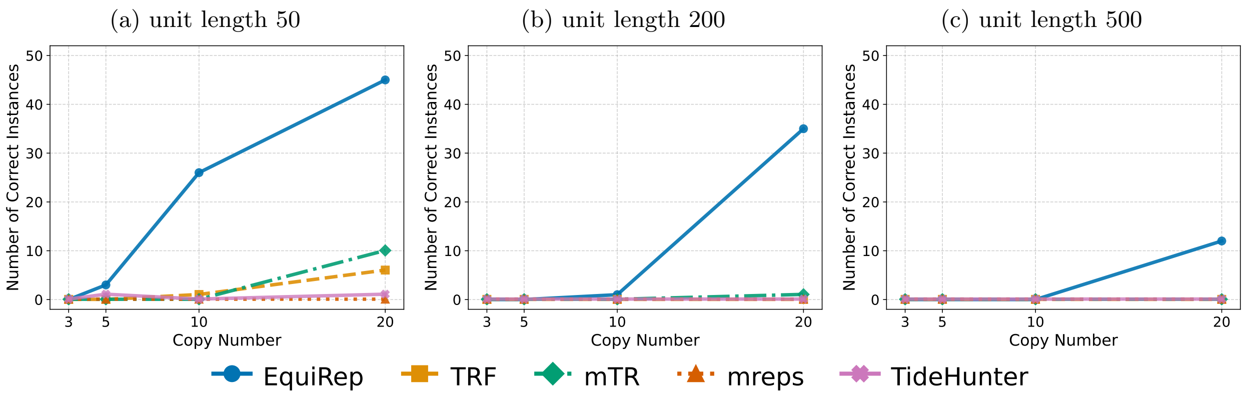

Supplementary Figure 10: Comparison of number of correct predictions on simulations with 3 recurring kmers at 20% error rate.

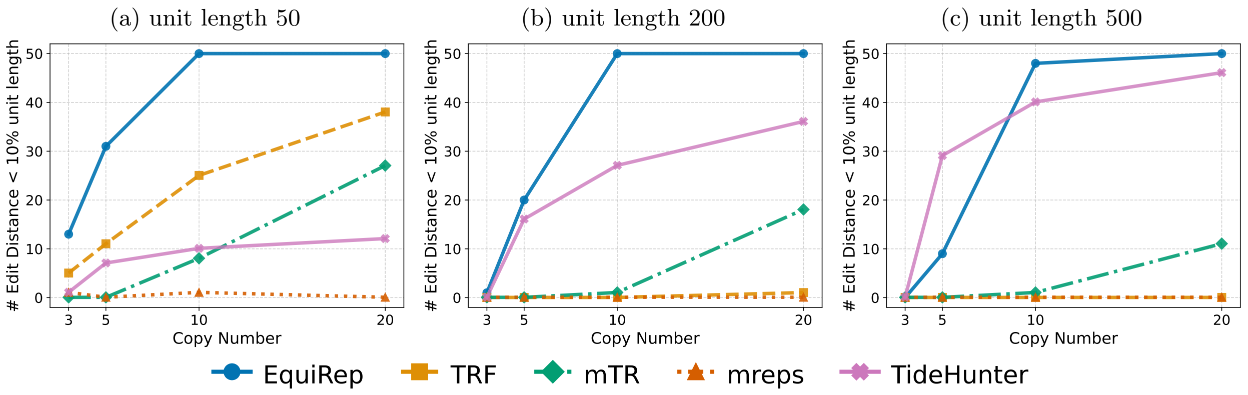

Supplementary Figure 11: Comparison of number of instances with edits less than 10% of the unit length on simulations with 3 recurring kmers at 20% error rate.

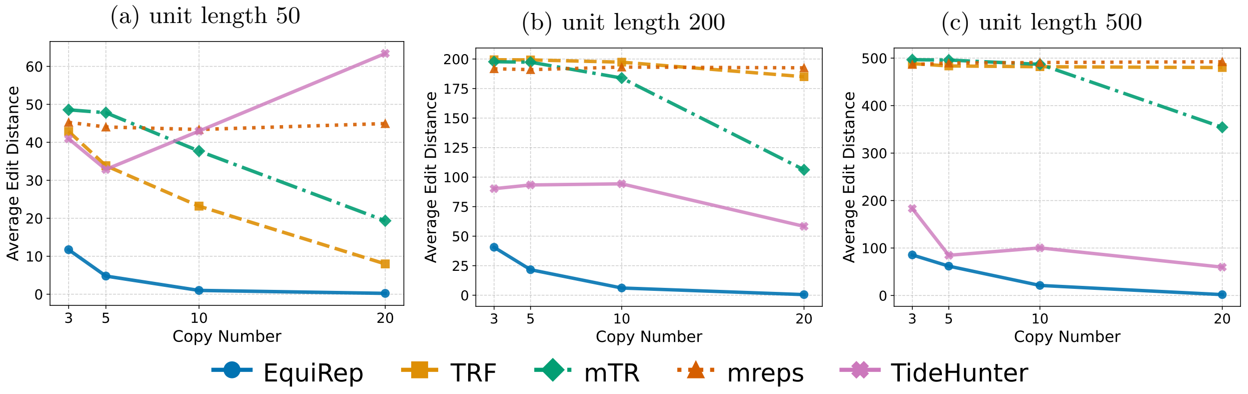

Supplementary Figure 12: Comparison of average edit distance on simulations with 3 recurring kmers at 20% error rate.

Supplementary Table 1: Comparison of running time in seconds fro different lengths and copy numbers on simulated data.

Length_CopyNumber	EquiRep	TRF	mTR	mreps	TideHunter
5_3	3.67	0.00	1.15	0.01	0.01
5_5	13.73	0.01	1.14	0.01	0.00
5_10	16.18	0.03	1.04	0.02	0.02
5_20	14.22	0.10	1.2	0.04	0.06
10_3	15.8	0.00	1.16	0.01	0.00
10_5	14.18	0.03	1.04	0.02	0.02
10_10	14.16	0.08	1.27	0.03	0.06
10_20	14.22	0.23	1.47	0.06	0.08
50_3	16.24	0.04	1.4	0.10	0.04
50_5	14.48	0.11	1.47	0.16	0.07
50_10	16.6	0.41	3.27	0.34	0.14
50_20	22.97	1.32	4.59	0.69	0.19
200_3	16.25	0.15	2.15	0.70	0.11
200_5	22.71	0.58	3.06	1.26	0.19
200_10	29.98	1.51	35.67	2.67	0.33
200_20	74.40	3.85	45.91	5.63	1.13
500_3	23.83	0.37	4.00	2.54	0.28
500_5	35.07	0.85	6.11	4.47	0.57
500_10	104.89	2.39	90.97	9.43	2.62
500_20	354.65	6.64	84.75	19.8	9.85
**Average**	41.9115	0.935	14.641	2.3995	0.7885
